# Molecular genetic characteristics of influenza A virus clinically isolated during 2011‐2016 influenza seasons in Korea

**DOI:** 10.1111/irv.12549

**Published:** 2018-03-30

**Authors:** Han Sol Lee, Ji Yun Noh, Joon Young Song, Hee Jin Cheong, Won Suk Choi, Hye Won Jeong, Seong‐Heon Wie, Woo Joo Kim

**Affiliations:** ^1^ Brain Korea 21 Plus for Biomedical Science College of Medicine Korea University Seoul Korea; ^2^ Division of Infectious Diseases Department of Internal Medicine Guro Hospital College of Medicine Korea University Seoul Korea; ^3^ Division of Infectious Diseases Department of Internal Medicine Ansan Hospital College of Medicine Korea University Ansan Korea; ^4^ Division of Infectious Diseases Department of Internal Medicine College of Medicine Chungbuk National University Cheongju Korea; ^5^ Division of Infectious Diseases Department of Internal Medicine St. Vincent's Hospital College of Medicine The Catholic University of Korea Suwon Korea

**Keywords:** hospital‐based influenza morbidity and mortality (HIMM), influenza A virus, substitution

## Abstract

**Background:**

The influenza virus is reportedly associated with 3‐5 million cases of severe illness and 250 000‐500 000 deaths annually worldwide.

**Objectives:**

We investigated the variation of influenza A virus in Korea and examined the association with death.

**Methods:**

A total of 13 620 cases were enrolled in the Hospital‐based Influenza Morbidity & Mortality surveillance system in Korea during 2011‐2016. Among these cases, a total of 4725 were diagnosed with influenza using RT‐PCR (influenza A; n = 3696, influenza B; n = 928, co‐infection; n = 101). We used 254 viral sequences from the 3696 influenza A cases for phylogenetic analysis using the BioEdit and MEGA 6.06 programs.

**Results:**

We found that the sequences of A/H3N2 in the 2011‐2012 season belong to subgroup 3C.1, whereas the sequences in the 2012‐2013 season pertain to subgroup 3C.2. The sequences in the 2013‐2014 and 2014‐2015 seasons involve subgroups 3C.3a and 3C.2a. The A/H1N1pdm09 subtype belongs to subgroup 6 and contains two clusters. In addition, sequence analysis confirmed the several substitutions of internal genes and gene substitutions associated with drug resistance (*I222V* in NA and *S31N* in M2) in the fatal cases. While statistical analysis found no significant associations between genetic differences in the viruses and mortality, mortality was associated with certain host factors, such as chronic lung disease.

**Conclusions:**

In conclusion, influenza A virus clade changes occurred in Korea during the 2011‐2016 seasons. These data, along with antigenic analysis, can aid in selecting effective vaccine strains. We confirmed that fatality in influenza A cases was related to underlying patient diseases, such as chronic lung disease, and further studies are needed to confirm associations between mortality and viral genetic substitutions.

## 1 INTRODUCTION

Influenza A viruses are classified under the *Orthomyxoviridae* family and consist of several subtypes, based on their hemagglutinin (HA) and neuraminidase (NA) composition. In some cases, reassortment of the 8 viral RNA segments may occur if different influenza viruses infect the same host, thereby generating new viral strains (antigenic shift). Mutations in the genes of influenza A viruses are enhanced by the absence of RNA proofreading enzymes; in particular, substitutions in the HA protein alter the viral antigenic epitopes sufficiently to avoid the host immune response (antigenic drift). These antigenic changes can trigger the generation of new strains and subgroups, leading to pandemics or new epidemics worldwide.^1^, ^2^, ^3^

Influenza A virus subtypes H3N2 (A/H3N2) and H1N1 (A/H1N1), and influenza B virus have been circulating in the human population every winter, and account for 3 to 5 million cases of severe illness and 250 000 to 500 000 deaths annually, mostly caused by secondary bacterial pneumonia in young children and the elderly.^4^, ^5^, ^6^, ^7^ To reduce the public health burden of influenza, the World Health Organization (WHO) recommends annual influenza vaccination and recommends the four strains to be included in vaccine composition. Seasonal vaccine strains are recommended twice a year because of the differences in the duration of winter in the Southern Hemisphere and Northern Hemisphere. Vaccine strain recommendations for effective influenza vaccination are based on antigenic analysis and viral genome data worldwide in addition to global surveillance systems of the circulating viruses. In addition, genome sequencing and analysis of circulating influenza viruses have provided comprehensive understanding of evolutionary models as well as epidemiologic insight based on analysis of antigenic determinants, drug resistance, and a variety of sequence‐based bioinformatics methods.^2^, ^8^, ^9^

Here, we analyzed the HA and NA genes of influenza A viruses prevalent in Korea during the 2011‐2016 seasons. These viruses were identified through the Hospital‐based Influenza Morbidity & Mortality (HIMM) surveillance system.^10^ In addition, we investigated the effect of mutations in the 8 segmented genes of influenza A viruses on risk of mortality for infected patients.

## 2 MATERIALS AND METHODS

### 2.1 Sample collection and viral isolation

Through the HIMM system, clinical and virological surveillance was conducted for patients visiting the emergency departments or being hospitalized due to influenza‐like illness at 10 university hospitals in Korea from 2011 to 2016 (n = 13 620). We obtained nasopharyngeal swab samples during the 2011‐2016 season and identified influenza viruses using RT‐PCR. Influenza A virus‐positive samples were identified as either influenza A/H3N2 or 2009 pandemic A/H1N1 (A(H1N1) pdm09) and subjected to sequencing for the determination of HA and NA genes. A total of 254 sequences were used in the phylogenetic study. Using influenza A virus‐positive samples, Madin‐Darby canine kidney (MDCK) cells were inoculated with severe acute respiratory infection (SARI) samples and cultured for 48‐72 h. The hemagglutination assay was performed to detect the influenza virus growth.^11^ Unfortunately, samples were not obtained for the 2011‐2013 seasons. Therefore, analysis of fatal cases was performed only for the 2013‐2016 seasons.

### 2.2 Viral RNA extraction and sequencing

Viral RNA was extracted using the QIAamp Viral RNA mini kit (Qiagen, Hilden, Germany) and then reverse‐transcribed using influenza A virus universal primers (Uni 12, 5′‐AGCAAAAGCAGG‐3′) with the Primescript 1st strand cDNA synthesis kit (Takara, Shiga, Japan). PCR amplification was carried out using primers specific for the viral RNA segments coding for HA (forward, 5′‐ AGCAAAAGCAGGGG‐3′; reverse, 5′‐AGTAGA AAC AAGGGTGTTTT‐3′), NA (forward, 5′‐AGCRAAAGCAGGRGTTTAAAA‐3′; reverse, 5′‐AGTAGAAACAAGGAGTTTTTT‐3′), nucleoprotein (NP; forward, 5′‐AGCRAAAGCAG GGTARATAAT‐3′; reverse, 5′‐AGTAGAAACAAGGGTATTTTT‐3′), nonstructural protein (NS; forward, 5′‐AGCRAAAGCAGGGTGACAAA‐3′; reverse, 5′‐AGTAGAAACAAGGGTGTTTTTTAT‐3′), matrix protein (M; forward, 5′‐AGCRAAAGCAGGTAGATATT‐3′; reverse, 5′‐AGTAGAAACAAGGTAGTT TTT‐3′), and RNA polymerase subunits PA (forward, 5′‐AGCRAAAGCAGGTACTGATYCGAAATG‐3′; reverse, 5′‐AGTAGAAACAAGGTACTTTTTTGGACA‐3′), PB1 (forward, 5′‐AGCRAAAGCAGGCA A ACCATTTGAATG‐3′; reverse, 5′‐AGTAGAAACAAGGCATTTTTTCATGAA‐3′), and PB2 forward, 5′‐AGCRAAAGCAGGTCAATTATATTCA‐3′; reverse, 5′‐AGTAGAAACAAGGTCGTTTTTAAACTA‐3′). After amplification, the sequence readouts of the PCR products were analyzed. The sequences obtained from this study were deposited in GenBank under accession no. KY063619 through KY063705, KY509553‐KY509793.

### 2.3 Sequence analysis

For the phylogenetic study, the sequences were compared with the NCBI‐registered full‐length nucleotide sequences of influenza A/H3N2 and A(H1N1) pdm09 viruses and vaccine strains (H1N1: A/California/07/2009; H3N2: A/Perth/16/2009, A/Victoria/361/2011, A/Texas/50/2012, and A/Switzerland/9715293/2013). The sequences were aligned using the BioEdit program. MEGA 6.06 was used to build the phylogenetic tree, using the maximum‐likelihood method by obtaining the initial tree for the heuristic search based on the neighbor‐joining method through a matrix of pairwise distances evaluated by the maximum composite likelihood approach.^12^ The bootstrap scores were set to 1000 (bootstrap values over 50 are shown above the tree branches).

### 2.4 Hemagglutination inhibition (HAI) assay

HAI titers were determined using standard procedures. In brief, antisera were pre‐treated overnight with receptor‐destroying enzyme (RDE) at 37°C and heat inactivated at 56°C for 30 min. Twofold serial dilutions of RDE‐treated antisera (50 μL) starting at a 1:10 dilution were incubated with 4 HA unit/25 μL of each virus and incubated at RT for 1 h. Next, 50 μL of 0.75% guinea pig red blood cells (gRBCs) was added, followed by 1‐h incubation at RT. We confirmed the coagulation of gRBCs and interpreted HAI results.

### 2.5 Neuraminidase inhibitor (NAI) assay

The NA‐Fluor^TM^ assay kit (Thermo Fisher Scientific, Waltham, MA, USA) was used according to the manufacturer′s protocol. Neuraminidase inhibitor was prepared in 10‐fold serial dilutions at 4× the final concentration in assay buffer (16.65 mm MES, 2 mm CaCl_2_, pH 6.5), and 25 μL was added to wells of a black flat‐bottom 96‐well microplate. Viral samples were diluted in assay buffer, and 25 μL was added to the NAI serial dilutions, and the samples were then incubated for 30 min at 37°C. NA‐Fluor Substrate (50 μL) was added to create a final assay concentration of 100 μm 4‐Methylumbelliferone sodium salt, and the samples incubated at 37°C for 1 h. No‐virus controls were included on each assay plate. Assay was terminated by addition of 100 μL of Stop Solution, and plates were read on a Victor 3 plate reader using Ex 355 nm/Em 460 nm settings. For data analysis, the relative fluorescence unit values of the no‐virus control wells were subtracted from the virus‐containing well values and data were processed using Graphpad^®^ Prism software.

### 2.6 Statistics analysis

To elucidate the factors associated with the death of influenza cases, fatal and non‐fatal cases were selected (1:3 ratio) on the collection date, sex, and age in the 2013‐2016 seasonal isolates. The A(H1N1)pdm09 fatal case sample was excluded because one fatal case was not appropriate for statistical analysis. The 2011‐2013 fatal case samples were excluded due to lack of appropriate non‐fatal case samples. Therefore, we analyzed the data pertaining to a total of 24 samples including 6 fatal and 18 non‐fatal cases in the 2013‐2016 season using Fisher's exact test. A *P*‐value <.05 was considered statistically significant.

## 3 RESULTS

### 3.1 HA and NA diversity of seasonal influenza A virus in Korea, 2011‐2016

A total of 13 620 patients were enrolled through the HIMM surveillance system during the 2011‐2016 seasons. These include 4725 patients positive for influenza A or B viruses with RT‐PCR, and 3696 (78.2%) of them were confirmed with influenza A virus, including subtype A(H1N1)pdm09 and H3N2 (Table 1). During this period, A/H3N2 was the predominant epidemic strain with the exception of 2015‐2016 season during which A/H1N1 co‐circulated. Because of this dominant pattern, we collected 43 sequences of A(H1N1)pdm09 in the 2012‐2014 and 2015‐2016 seasons, and 211 sequences of A/H3N2 in the 2011‐2015 seasons. A phylogenetic tree was constructed to confirm the substitution patterns of HA and NA genes in the influenza A virus.

**Table 1:** Influenza types in Korea during the 2011‐2016 seasons based on RT‐PCR

Seasons	Number of cases enrolled in HIMM	RT‐PCR			Influenza type			Number of samples analyzed	
		Positive	Negative	Non‐sample	FluA	FluB	Co‐infection	A/H1N1	A/H3N2
2011‐2012	2252	927 (41.1%)	1243 (55.2%)	82 (3.6%)	731 (78.9%)	182 (19.6%)	14 (1.5%)	‐	2
2012‐2013	1667	681 (40.9%)	882 (52.9%)	104 (6.2%)	676 (99.3%)	4 (0.6%)	1 (0.1%)	1	93
2013‐2014	3082	1295 (42.0%)	1328 (43.1%)	459 (14.9%)	978 (75.5%)	307 (23.7%)	10 (0.8%)	22	46
2014‐2015	3901	1298 (33.3%)	2002 (51.3%)	601 (15.4%)	962 (74.1%)	273 (21.0%)	63 (4.9%)	‐	70
2015‐2016	2718	524 (19.3%)	1543 (56.8%)	651 (24.0%)	349 (66.6%)	162 (30.9%)	13 (2.5%)	20	‐
Total	13 620	4725 (34.7%)	6998 (51.4%)	1897 (13.9%)	3696 (78.2%)	928 (19.6%)	101 (2.1%)	43	211

**Figure 1:**
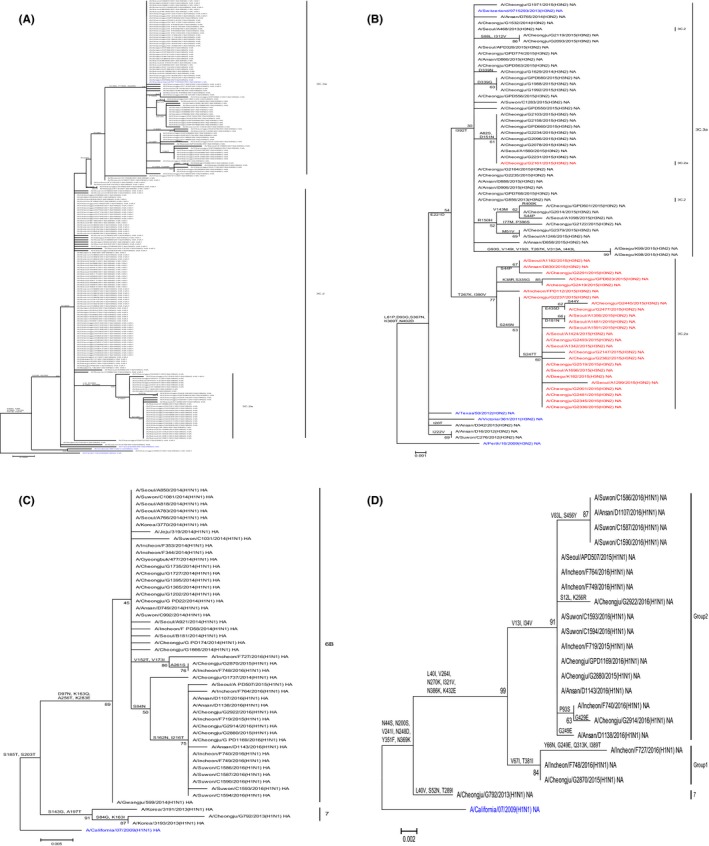
Phylogenetic tree of HA and NA genes of influenza A virus in Korea during the 2011‐2016 seasons. The (A) HA and (B) NA sequences of A/H3N2 isolate in Korea showed clustering in the phylogenetic tree. The sequences of clade 3C.2a in HA tree are shown in red font (B) NA of A/H3N2. The phylogenetic tree of A/H1N1 isolate (C) HA and (D) NA sequences reveals distinct groups. The phylogenetic tree was inferred from the recommended H1N1 vaccine strains (A/California/07/2009), H3N2 vaccine (A/Perth/16/2009, A/Victoria/361/2011, A/Texas/50/2012, A/Switzerland/9715293/2013), and clinical isolates identified in Korea (2011‐2016). The font colors represent the vaccine strain (blue)

The HA phylogenetic tree was developed using 43 HA sequences of A(H1N1)pdm09, 211 HA sequences of A/H3N2 and the recommended vaccine strains (A/Perth/16/2009, A/Victoria/361/2011, A/Texas/50/2012, A/Switzerland/9715293/2013, and A/California/07/2009). In Figure 1, most of the A/H1N1 sequences clustered in clade 6B with the exception of A/Cheongju/G792/2013. The clade 6B was differentiated into two groups by the amino acid substitutions V152T and V173I in group 1 and S84N, S162N, and I216T in group 2. The phylogenetic tree of A(H1N1)pdm09 NA genes has shown that the two groups varied in terms of amino acid substitutions V67I, T381I in group 1 and V13I, I34V in group 2. These groups were generally congruent in the A(H1N1)pdm09 HA and NA phylogenetic tree. In the HA phylogenetic tree of A/H3N2, the viruses could be categorized into clades 3C.2 and 3C.3. Specifically, viruses from the 2012‐2013 and 2013‐2014 seasons clustered in clades 3C.2 and 3C.3a, respectively. Viruses from the 2014‐2015 season could be split into two clades based on the amino acid substitutions A138S, F159S, and N225D for clade 3C.3a and L3I, N144S, F159Y, and N225D for clade 3C.2a. The antigenic characterization was performed to determine whether the HA sequence substitution of A/H3N2, which indicates clade classification, is indicative of antigenic variation. We also confirmed significant antigenic changes in influenza A virus as a result of genetic variation in influenza A/H3N2 viruses (Table 2).

**Table 2:** Antigenic characterization of H3N2 influenza A viruses

Reference viruses		Post‐infection sheep antisera			Genetic clade
		A/Victoria/361/2011	A/Texas/50/2012	A/Switzerland/9715293/2013	
A/H3N2 vaccine strains		>1280	>1280	>1280	
Test viruses					
2013‐2014 season isolates	A/Ansan/D765/2014	640	320	1280	3C.3a
	A/Cheongju/G1532/2014	320	160	640	3C.3a
	A/Cheongju/G1629/2014	640	320	1280	3C.3a
	A/Ansan/D799/2014	320	160	1280	3C.3a
	A/Cheongju/G1739/2014	640	320	1280	3C.3a
2014‐2015 season isolates	A/Cheongju/G2156/2015	>1280	1280	>1280	3C.3a
	A/Suwon/K98/2015	160	80	1280	3C.3a
	A/Ansan/D858/2015	320	160	1280	3C.3a
	A/Ansan/D906/2015	320	160	1280	3C.3a
	A/Cheongju/G1971/2015	160	80	>1280	3C.3a
	A/Cheongju/G2103/2015	640	320	1280	3C.3a
	A/Seoul/A1284/2015	1280	1280	1280	3C.2a
	A/Seoul/A1251/2015	1280	640	1280	3C.2a
	A/Seoul/A1342/2015	80	40	80	3C.2a
	A/Ansan/D830/2015	160	80	160	3C.2a
	A/Seoul/A1299/2015	160	40	80	3C.2a
	A/Cheongju/G2477/2015	80	40	160	3C.2a

Based on the NA phylogenetic tree, viruses from the 2014‐2015 season fell into two distinct clusters based on amino acid substitution I392T for clade 3C.3a and T267K and I380V for clade 3C.2a. Interestingly, these differences in the NA cluster were consistent with differences in the HA clusters 3C.2a and 3C.3a, except for strains A/Cheongju/G2161/2015(H3N2), A/Seoul/A468/2013(H3N2), and A/Cheongju/G856/2013(H3N2).

### 3.2 Genetic analysis of 8 segmented genes in the fatal influenza A virus isolates

Viral virulence can be increased by mutating non‐structural proteins (Table S1). Therefore, internal gene sequences from influenza A viruses from fatal cases were used in the genetic analysis. Genetic analysis of 8 segments (HA, NA, M, NP, NS, PA, PB1, and PB2) was performed using sequences of fatal isolates (A(H1N1)pdm09; n = 1, A/H3N2; n = 11) and vaccine strains. Most of the fatal cases had underlying conditions, such as diabetes, chronic respiratory diseases, and chronic medical illnesses (Table 3).

**Table 3:** Clinical and laboratory characteristics of the 12 fatal cases of influenza

Season	Isolated virus	Age/sex	BMI	Body temperature at presentation (°C)	Pre‐existing condition	Cause of death	Microbiologic test			Antiviral drug treatment (days from symptom onset to administration of antiviral)
							Sputum culture	Blood culture	Pneumococcal urinary antigen test	
2011‐2012	A/Ansan/D16/2012 (H3N2)	78/M	27.5	None	Diabetes, hypertension, chronic cerebrovascular disease	Pneumonia	*Streptococcus pneumoniae*	Not done	Positive	Oseltamivir 75 mg bid (0) plus
										Peramivir 300 mg qd (1)
	A/Suwon/C276/2012 (H3N2)	89/M	24.2	37.3	Diabetes, hypertension, chronic respiratory diseases, BPH	Exacerbation of COPD	MRSA	Not done	Negative	Oseltamivir 75 mg bid (1)
2012‐2013	A/Seoul/A468/2013 (H3N2)	89/M	23.7	37.6	None	Multi‐organ failure	*Streptococcus pneumoniae*	Not done	Positive	Peramivir 100 mg qd (5)
	A/Cheongju/G792/2013 (H1N1)	74/F	15.2	38	Cancer	Pneumonia	Normal flora	Not done	Positive	Oseltamivir 150 mg bid (1) plus
										Peramivir 300 mg qd (3)
	A/Ansan/D342/2013 (H3N2)	72/M	20.7	38.6	Chronic cardiovascular disease, AML	Pneumonia	*Pseudomonas aeruginosa*	*Pseudomonas aeruginosa*	Negative	Oseltamivir 75 mg bid (2) plus
										Peramivir 250 mg qd (2)
	A/Cheongju/G856/2013 (H3N2)	93/F	20	Non	Chronic cerebrovascular disease	Pneumonia	No growth	Not done	Negative	Oseltamivir 75 mg bid (1)
2013‐2014	A/Ansan/D765/2014 (H3N2)	80/M	23.1	38.6	Chronic respiratory diseases, COPD	Pneumonia, sepsis	Normal flora	Not done	Not done	Oseltamivir 75 mg bid (1)
	A/Cheongju/G1532/2014 (H3N2)	86/F	17.8	36.8	Hypertension	Pneumonia, sepsis	Normal flora	*Staphylococcus aureus*	Negative	Oseltamivir 75 mg bid (1)
	A/Cheongju/G1629/2014 (H3N2)	86/M	16.5	37.5	COPD	Pneumonia	MRSA	Not done	Negative	Oseltamivir 75 mg bid (7)
2014‐2015	A/Seoul/A1284/2015 (H3N2)	97/F	20.2	37.8	Diabetes, chronic respiratory diseases, anemia, dementia, osteoporosis	Pneumonia	*Pseudomonas fluorescens*	Not done	Negative	Peramivir 300 mg qd (1)
	A/Seoul/A1251/2015 (H3N2)	92/M	17.9	38.2	Diabetes, asthma, chronic respiratory diseases, TB, BPH	Pneumonia, sepsis	MRSA, *Escherichia coli*	Not done	Negative	Peramivir 300 mg qd (2)
	A/Cheongju/G2156/2015 (H3N2)	83/F	17.3	None	Hypertension, chronic respiratory diseases	Pneumonia	Not done	Not done	Negative	Oseltamivir 75 mg bid (3)

BMI, body mass index; BPH, benign prostatic hyperplasia; COPD, chronic obstructive pulmonary disease; TB, tuberculosis; AML, acute myeloid leukemia; MRSA, methicillin‐resistant *Staphylococcus aureus*.

Compared with vaccine strain, amino acid mutations were found in the A/Cheongju/G792/2013(H1N1) HA gene: the P83S, S84G, S143G, K163I, G170R, S185T, A197T, S203T, A261T, G262E, H273N, and I321V substitutions were located in the HA1 region, the E47K, S124N, I183V, and V193A substitutions were located in the HA2 region (Table 4).

**Table 4:** Amino acid substitutions in the 8 segments of influenza strains isolated in Korea during 2011‐2015 seasons compared to the vaccine strains

Subtype	Seasons	Sample name	HA	NA	M1	M2	NP	NS1	NS2	PA	PB1	PB2	Genetic clade (HA based)
H3N2	H3N2 Vaccine	A/Victoria/361/2011	‐	‐	‐	‐	‐	‐	‐	‐	‐	‐	
	2011‐2012	A/Suwon/C276/2012	Q33R, E50K, V186G, D188G, R255T, P273R, N278K, ***I149T***	I222V, K258E, T329N	‐	V51I,	S359P, M374I, M440V	D209N, K229E	‐	E351D, R356K, M407I	D581N	V606A	3C.1
		A/Ansan/D16/2012	Q33R, V186G, N278K	I222V, K258E, T329N	‐	G16E, V51I, E97G	V197A, M374I	V22F, D209N, K229E	K39R	E351D, M407I	‐	M66T, R389K	3C.1
	2012‐2013	A/Ansan/D342/2013	Q33R, N145S, V186G, N278K	I20T, K258E, T329N	‐	‐	K470M	E26K, K78R, D209N, K229E	‐	M407I	R361K	Y55F, R194Q, L384I	3C.2
		A/Seoul/A468/2013	Q33R, N145S, V186G, N246Y, N278K, ***D160N***	E221D, K258E, T329N, I392T	‐	‐	K357R	E26K, I182V, D209N, K229E	‐	M407I, V668I, N675K	M317T	‐	3C.2
		A/Cheongju/G856/2013	Q33R, N145S, V186G, N278K, ***D160N***	E221D, K258E, T329N, I392T	‐	‐	‐	E26K, I182V, D209N, K229E	‐	G240V, M407I, V668I, N675K	‐	‐	3C.2
	H3N2 Vaccine	A/Switzerland/9715293/2013	Q33R, T128A, A138S, R142G, N145S, F159S, V186G, N225D, N262K, K326R	E221D, K258E, T329N, I392T	‐	‐	S217G	E26K, M124I, D209N, K229E	‐	Q256K, I308V, I554V, K605R, V669I, H713Y	‐	‐	3C.3a
	2013‐2014	A/Cheongju/G1629/2014	Q33R, T128A, A138S, R142G, N145S, F159S, V186G, N225D, N262K, K326R	E221D, K258E, T329N, D339N, I392T	‐	‐	S217G, S482N	E26K, M124I, D209N, K229E	‐	Q256K, I308V, I554V, K605R, V669I	‐	‐	3C.3a
		A/Ansan/D765/2014	Q33R, T128A, A138S, R142G, N145S, F159S, V186G, N225D, N262K, K326R, ***V73A***	Y67F, E221D, K258E, T329N, I392T	‐	‐	S217G	E26K, M124I, D209N, K229E	‐	Q256K, I308V, I554V, K605R, V669I	R52K, L424M	V338I, S709N	3C.3a
		A/Cheongju/G1532/2014	Q33R, T128A, A138S, R142G, N145S, F159S, V186G, N225D, N262K, K326R	E221D, K258E, T329N, I392T	‐	‐	S217G	E26K, M124I, A202T, D209N, K229E	‐	Q256K, I308V, A455G, I554V, K605R, V669I	T400K	A559V, S643T	3C.3a
	2014‐2015	A/Cheongju/G2156/2015	Q33R, T128A, A138S, R142G, N145S, F159S, V186G, A212S, N225D, N262K, K326R, ***A201V***	E221D, K258E, T329N, I392T	‐	‐	I136L, S217G	E26K, M124I, D209N, K229E	‐	Q256K, I308V, D396E, I554V, K605R, V669I	‐	R390K, M476L	3C.3a
		A/Seoul/A1284/2015	L3I, Q33R, N144S, N145S, F159Y, V186G, N225D, N278K, Q311H, ***D160N***	E221D, S245N, S247T, K258E, T267K, T329N, I380V	‐	I39M	M481I	E26K, D209N, K229E	‐	N272S, D396E, M407I, V668I, N675K	‐	V63I, I589T	3C.2a
		A/Seoul/A1251/2015	L3I, Q33R, N144S, N145S, F159Y, K160T, V186G, N225D, N278K, Q311H, ***D160N***	D151N, E221D, S245N, S247T, K258E, T267K, T329N, I380V, W383C	‐	I39M	M481I	E26K, D209N, K229E	‐	N272S, D396E, M407I, V668I, N675K	W580C	V63I, I589T	3C.2a
H1N1	H1N1 Vaccine	A/California/07/2009	‐	‐	‐	‐	‐	‐	‐	‐	‐	‐	
	2012‐2013	A/Cheongju/G792/2013	P83S, S84G, S143G, K163I, G170R, S185T, A197T, S203T, A261T, G262E, H273N, I321V, ***E47K, S124N, I183V, V193A***	L40V, N44S, S52N, N200S, V241I, N248D, T289I, Y351F, N369K	V80I, M192V, K230R	D21G	V100I, L108I	L90I, I111T, V117M, I123V, N205S	T48A	I30T, Y161F, P224S, E252V, N321K, A343T, V407I, R673S	G154D, I397M, I435T, H456Y	D195N, R293K, V344M, I354L, V731I	7

HA2 domain is indicated in bold‐italic font

Compared with the NA gene of the vaccine strain, A/Cheongju/G792/2013(H1N1) carried L40V, N44S, S52N, N200S, V241I, N248D, T289I, Y351F, and N369K substitutions. In addition, several substitutions were identified in the other 6 segments (Table 4).

In the A/H3N2 analysis, we confirmed variations for the cluster differences (3C.2a and 3C.3a) in the *HA* and NA genes. In addition, the NA sequence of the 2011‐2012 season (n = 2) carried an I222V substitution, an NA inhibitor resistance mutation.^13^, ^14^, ^15^, ^16^ The NA sequence of several samples carried substitutions: I20T in A/Ansan/D342/2013, D339N in A/Cheongju/G1629/2014, Y67F in A/Ansan/D765/2014, and D151N, W383C in A/Seoul/A1251/2015. The NAI assay was conducted using vaccine strains and viruses isolated in the 2013‐2016 seasons to determine whether NA gene mutations affected virulence due to antiviral resistance. The NAI assay demonstrated the absence of resistant viruses in the fatal influenza cases (Table 5).

**Table 5:** Neuraminidase inhibition of fatal case isolates

Sample	2013‐2014 season			2014‐2015 season			A/Switzerland/9715293/2013
	D765	G1532	G1629	G2156	A1284	A1251	
IC_50_ (nm)	0.275	0.283	0.279	0.245	0.155	0.210	0.218
95% CI	0.259‐0.292	0.266‐0.300	0.267‐0.292	0.224‐0.269	0.147‐0.163	0.192‐0.230	0.204‐0.232

In the analysis of internal genes, the M sequence of all the isolates investigated harbored the S31N genetic marker for adamantine resistance in M2. By comparing the sequences from each season with the A/H3N2 vaccine strain (A/Victoria/361/2011), we found several amino acid substitutions (Table 4). Interestingly, the PA gene of A/Ansan/D765/2014, A/Cheongju/G1532/2014, A/Cheongju/G1629/2014, A/Cheongju/G2156/2015, and A/Switzerland/ 9715293/2013 carried 5 amino acid mutations: Q256K, I308V, I554V, K605R, and V669I. These PA substitutions were consistent with clade 3C.2a in the HA phylogenetic tree. As shown in Table 4, we confirmed that the substitutions of NA, NS1, and PA coincided with HA‐based genetic clade. In addition, the PB1 and PB2 genes revealed multiple non‐synonymous substitutions (Table S2).

Statistical analysis was performed to study the association between amino acid substitution and death in fatal cases (Table 6). Although some substitutions (I39M of M2 and M481I of NP) showed a *P* value of .054, they were not significant in the correlation analysis of genetic substitution and mortality. As shown in Table 7, our correlation analysis revealed that chronic lung disease was more frequently associated with fatal than non‐fatal cases.

**Table 6:** Amino acid substitutions associated with mortality based on Fisher's exact test

Substitutions	Fatal cases (n = 6)	Non‐Fatal cases (n = 18)	Fisher's *P*‐value
M2_I39M	2	0	.054
NP_I136L	1	4	1.000
NP_S217G	4	15	.568
NP_M481I	2	0	.054
NP_S482N	1	0	.250
NS1_M124I	4	15	.568
NS1_A202T	1	0	.250
PA_Q256K	4	15	.568
PA_N272S	2	3	.568
PA_I308V	4	15	.568
PA_M407I	2	3	.568
PA_A455G	1	0	.250
PA_I554V	4	15	.568
PA_K605R	4	15	.568
PA_V668I	2	3	.568
PA_V669I	4	15	.568
PA_N675K	2	3	.568
PB1_R52K	1	0	.250
PB1_T400K	1	0	.250
PB1_L424M	1	0	.250
PB1_W580C	1	0	.250
PB2_V63I	2	4	.618
PB2_V338I	1	1	.446
PB2_R390K	1	4	1.000
PB2_M476L	1	4	1.000
PB2_A560V	1	0	.250
PB2_I589T	2	3	.568
PB2_S644T	1	0	.250
PB2_S710N	1	0	.250

**Table 7:** Demographic characteristics of patients with influenza

	Fatal cases (n = 6)	Non‐fatal cases (n = 18)	*P* value^a^
Age (year), mean ± SD	87.3 ± 6.2	82.0 ± 7.1	.12
Sex (male), n (%)	3 (50.0)	9 (50.0)	1
Comorbidity	4 (66.7)	14 (77.8)	.62
Diabetes mellitus	2 (33.3)	5 (27.8)	1
Cardiovascular disease	‐	5 (27.8)	.28
Cerebrovascular disease	‐	6 (33.3)	.28
Neuromuscular disease	‐	4 (22.2)	.54
Chronic lung disease	4 (66.7)	2 (11.1)	.02
Asthma	1 (16.7)	2 (11.1)	1
Chronic kidney disease	‐	1 (5.6)	1
Chronic liver disease	‐	3 (16.7)	.55
Malignancy	‐	1 (5.6)	1

a Fisher's exact test.

In conclusion, statistical analysis showed no significant association between the viral genetic differences and mortality; however, the mortality was increased by host factors such as chronic lung disease.

## 4 DISCUSSION

We performed sequencing and phylogenetic analysis of influenza A viruses collected from patients during the 2011‐2016 season in Korea, to verify previously known and novel virulence factors in patients with fatal influenza and the evolution of influenza viruses with time. We confirmed the genetic changes among the influenza A viruses during 2011‐2016 in Korea. The A(H1N1)pdm09 in 2015‐2016 was grouped into clade 6B and separated into two clusters by substitution V152T, V173I in group 1 and S84N, S162N, and I216T in group 2 over time. Interestingly, the differences of HA clusters were consistent with the *NA* genes. In the A/H3N2, the sequences in the 2013‐2015 season carried substitutions: A138S, F159S, N225D, N241D, and K326R in HA1 or N144S, F159Y, N225D, and Q311H in HA1, and were categorized into clade 3C.3a and 3C.2a. In addition, the *NA* genes were distinguished into two clusters: I392T in cluster 1 and T267K, I380V in the other cluster. Interestingly, these results were congruent with *HA* clustering: 3C.3a and 3C.2a. Further, sequences detected in the 2012‐2013 season (HA clade 3C.2) showed I392T substitution in the *NA* gene. These mutations were generated earlier than the substitution of HA clade 3C.3a. Previous studies recognized several antigenic sites (A–E) and receptor‐binding sites (RBS) in the *HA* gene of A/H3N2.^2^, ^17^, ^18^, ^19^, ^20^ Several researchers reported that antigenic drift was caused by single amino acid substitutions near the RBS of the influenza A virus.^2^, ^20^, ^21^ The HA proteins of 3C.2a and 3C.3a viruses are substituted around the RBS, particularly D225N, 126NWT/AG, and 144NN/SSF in HA1, compared with A/Victoria/361/2011. These mutations in the RBS of HA are associated with changes in the protein surface, electrostatic charge, and N‐linked glycosylation. Based on the results of antigenic characterization, we suggest that the isolates belonging to the 3C.3a clade included the A/Switzerland/297135/2013–like virus, which carried a twofold HAI titer of the A/Switzerland/ virus. In addition, the isolates in the 3C.2a clade differ from the vaccine strain in antigenicity due to the 8‐ to 32‐fold differences from the vaccine strain in the HAI titer.

This difference seems to be due to structural variation in HA from genetic substitution. Our study demonstrated the variation patterns of epidemic influenza viruses in Korea using genetic as well as antigenic analyses. These data can be used to select vaccine strains and to analyze virus substitution patterns over time.

H3N2 seasons become increasingly severe, with higher numbers of hospitalizations and deaths. We studied the molecular genetics of influenza A viruses and factors associated with patient death following infection with influenza A/H3N2 during the 2011‐2016 seasons in Korea. First, we identified genetic substitutions in viruses from fatal cases. The I222V substitution in the NA protein was found in only the two A/H3N2 viruses from the 2011‐2012 season. However, other antidrug mutations, H274Y (N2 numbering) and I119V, were not confirmed. Previous studies have shown that the single I222V/M substitution in the NA protein is associated with marginal levels of resistance to oseltamivir, while synergistically increased drug resistance was associated with E119V and H274Y substitutions.^15^, ^16^, ^19^, ^22^, ^23^, ^24^, ^25^, ^26^ The S31N substitution in the M2 protein was frequently detected in the more recent viral sequences and reference sequences.^27^ In addition, V51I and I39M substitutions were identified in the 2011‐2012 and 2014‐2015 fatal case sequences. Among these two substitutions, V51I may enhance the fitness of M2 protein to increase the frequency of adamantine resistance associated with S31N mutation and the substitution of V51‐affected viral replication.^28^ The I39 of M2 was located in the transmembrane region, and substitution of the transmembrane region could affect M2 function, aiding in resistance to M2 inhibitors and transport to the cell surface. In the NP sequences, M374I and M481I were identified only in fatal case sequences. These substitutions have been reported to be involved a T‐cell epitope presented by MHC molecules.^29^, ^30^ In addition, it was previously reported that other substitutions were identified in the A/Cheongju/G792/2013(H1N1) (T48A in NS2) and in the A/Seoul/A468/2013(H3N2) (K357R in NP). The T48A in NS2 of A(H1N1)pdm09 contributed to the enhanced type I IFN antagonistic property of A/Vietnam/UT3062/04, leading to high virulence in ferrets. Mutations in amino acid 357 of NP, which is in the PB2 binding region of NP protein, have been reported that be involved in a shift in host specificity (Q in avian and K in human).

Statistical analysis was performed to confirm the association between amino acid substitution and death in fatal cases. Unfortunately, statistical analysis was conducted only for the 2013‐2015 season using appropriate controls (non‐fatal case samples). The I39M substitution of M2 and the M481I substitution of NP may be weakly correlated based on the *P*‐value of .054. Interestingly, the I39M of M2 and M482I of NP were identified in the same isolates (A/Seoul/A1284/2015, A/Seoul/A1251/2015). These internal proteins are involved in a variety of host responses and associated with the infectivity and replication of viruses; therefore, substitutions in these proteins can have a variety of effects.^31^, ^32^ A significant association between underlying diseases in patients and mortality revealed a significant correlation with chronic lung disease, confirming a well‐known relationship.

In conclusion, we observed clade changes in influenza A viruses in Korea from 2011 to 2016. Prediction of clade change using bioinformatics analysis of these data, along with antigenic analyses, can help select effective vaccine strains. We confirmed that the severity of influenza A virus infection was related to underlying patient diseases, such as chronic lung disease, and further studies are needed to confirm associations between mortality and genetic substitutions in the viruses.
